# Point Cloud Quality Assessment Using a One-Dimensional Model Based on the Convolutional Neural Network

**DOI:** 10.3390/jimaging10060129

**Published:** 2024-05-27

**Authors:** Abdelouahed Laazoufi, Mohammed El Hassouni, Hocine Cherifi

**Affiliations:** 1 Research Laboratory in Computer Science and Telecommunications (LRIT), Faculty of Sciences, Mohammed V University in Rabat, Rabat 1014, Morocco; 2 Faculty of Letters and Human Sciences, Mohammed V University in Rabat, Rabat 8007, Morocco; mohamed.elhassouni@flsh.um5.ac.ma; 3 Carnot Interdisciplinary Laboratory of Burgundy (ICB) UMR 6303 CNRS, University of Burgundy, 21000 Dijon, France

**Keywords:** point cloud, NR metric, convolutional neural network (CNN), transfer learning

## Abstract

Recent advancements in 3D modeling have revolutionized various fields, including virtual reality, computer-aided diagnosis, and architectural design, emphasizing the importance of accurate quality assessment for 3D point clouds. As these models undergo operations such as simplification and compression, introducing distortions can significantly impact their visual quality. There is a growing need for reliable and efficient objective quality evaluation methods to address this challenge. In this context, this paper introduces a novel methodology to assess the quality of 3D point clouds using a deep learning-based no-reference (NR) method. First, it extracts geometric and perceptual attributes from distorted point clouds and represent them as a set of 1D vectors. Then, transfer learning is applied to obtain high-level features using a 1D convolutional neural network (1D CNN) adapted from 2D CNN models through weight conversion from ImageNet. Finally, quality scores are predicted through regression utilizing fully connected layers. The effectiveness of the proposed approach is evaluated across diverse datasets, including the Colored Point Cloud Quality Assessment Database (SJTU_PCQA), the Waterloo Point Cloud Assessment Database (WPC), and the Colored Point Cloud Quality Assessment Database featured at ICIP2020. The outcomes reveal superior performance compared to several competing methodologies, as evidenced by enhanced correlation with average opinion scores.

## 1. Introduction

Recently, the utilization of 3D models has seen a significant expansion in various fields, including virtual and mixed reality, computer-aided diagnosis, architecture, and the preservation of cultural heritage. However, when these 3D models undergo operations such as simplification and compression, they can potentially introduce different distortion types that negatively impact the visual quality of 3D point clouds. To tackle this problem, there is a growing demand for robust methods to evaluate perceived quality. Traditionally, assessing distortion levels in 3D models has depended on human observers, which is time-consuming and resource-intensive. To streamline this, objective methods have emerged as a practical solution [1]. These methods involve the implementation of automated metrics that aim to replicate the judgments of an ideal human observer. These metrics can generally be categorized into three groups: full reference (FR) [2,3,4,5,6], reduced reference (RR) [7,8], and no-reference (NR) [9,10,11,12,13,14,15]. Among these, blind methods, which do not rely on reference models, have gained particular significance, especially in real-world applications [16,17,18,19].

Three-dimensional point cloud is a collection of points, each characterized by geometric coordinates and potentially additional attributes such as color, reflectance, and surface normals.

Unlike 2D media, such as images and videos, which are organized in a regular grid, the points in 3D point clouds are scattered throughout space. Therefore, there is a need to explore methods for extracting effective features from these scattered points to assess quality.

To date, only a limited set of metrics for assessing the quality of point clouds without reference, known as NR-PCQA (No-Reference Point Cloud Quality Assessment), have been developed. Chetouani et al. [20] adopted an approach involving extracting hand-crafted features at the patch level and using traditional CNN models for quality regression. PQA-net [9] employs multi-view projection as a method for feature extraction. Zhang et al. [13] took a distinct approach by using various statistical distributions to estimate quality-related parameters based on the distributions of geometry and color attributes. Fan et al. [21] focus on inferring the visual quality of point clouds through the analysis of captured video sequences. Liu et al. [10] utilized an end-to-end sparse CNN to predict quality. Yang et al. [22] extended their efforts by transferring quality information from natural images to enhance the understanding of the quality of point cloud rendering images, employing domain adaptation techniques.

Recently, Convolutional Neural Networks (CNNs) have emerged as the predominant choice for various Computer Vision and Machine Learning tasks. CNNs are feedforward Artificial Neural Networks (ANN) characterized by their convolutional and subsampling layer arrangement. Deep 2D CNNs, with numerous hidden layers and millions of parameters, can learn intricate objects and patterns, particularly when trained on extensive visual datasets with ground-truth labels. When properly trained, this capability positions them as the primary tool for various engineering applications that involve 2D signals, such as images and video frames.

However, this strategy may not always be feasible for numerous applications dealing with 1D signals, particularly when the training dataset is constrained to a specific application. To address this challenge, 1D CNNs have been recently introduced and have rapidly demonstrated cutting-edge performance across multiple domains. These domains include personalized biomedical data classification and early diagnosis, structural health monitoring, anomaly detection, identification in power electronics, and detecting faults in electrical motors. Among the technical applications of the 1D CNNs we quote automatic speech recognition, vibration-based structural damage detection in civil infrastructure, and real-time electrocardiogram monitoring [23].

Despite the growing importance of 1D CNNs in various applications, there exists a current void in the literature regarding point cloud quality assessment using these networks. In this context, we introduce a novel method in this paper for evaluating the visual quality of 3D point clouds. Our method revolves around a transfer learning model grounded in a one-dimensional CNN architecture. The main contributions of this paper are summarized as follows:

- The introduction of a novel methodology that adapts a one-dimensional Convolutional Neural Network (1DCNN) architecture for evaluating the visual quality of 3D point clouds.
- The design of the 1DCNN network tailored for point clouds by transforming a 2D CNN model into a 1D variant.
- The incorporation of transfer learning using a pre-trained ImageNet model to initialize the 1DCNN network for point cloud quality evaluation.

The rest of this paper is structured as follows: Section 2 provides an overview of the related work, Section 3 introduces the proposed method, Section 4 presents the experimental setup and the results of a comparative evaluation against alternative solutions, and finally, Section 5 concludes the paper.

## 2. Related Work

In the literature, the most current PCQA approaches can be broadly grouped into point-based, feature-based, and projection-based methods. A point-based quality metric directly compares the geometry or characteristics between the reference and distorted point clouds, assessing them point by point and establishing necessary point correspondences. The Point-to-Point (Po2Po) [24] and Point-to-Plane (Po2Pl) [25] metrics stand out as the most popular point-based geometry quality evaluation methods. In the Po2Po metric, each point in a degraded or reference point cloud is matched with its nearest corresponding point in the opposite cloud, and subsequently, the Hausdorff distance or Mean Squared Error (MSE) distance is computed for all point pairs. One significant limitation of these metrics is their failure to consider that point cloud points represent the surfaces of objects in the visual scene. Tian et al. [25] introduced Point-to-Plane (Po2Pl) metrics to address this issue. These metrics represent the underlying surface at each point as a plane perpendicular to the normal vector at that specific point. This approach yields smaller errors for points closer to the point cloud’s surface, which is modeled as a plane. Currently, the MPEG-endorsed point cloud geometry quality metrics include Po2Po and Po2Pl and their corresponding Peak Signal-to-Noise Ratio (PSNR) [5]. In addition, Alexiou et al. [26] proposed a Plane-to-Plane (Pl2Pl) metric, which evaluates the similarity between the underlying surfaces associated with corresponding points in the reference and degraded point clouds. In this scenario, tangent planes are estimated for both the reference and degraded points, and the angular similarity between them is examined.

In their work [27], Javaheri et al. introduced a geometry quality metric that relies on the Generalized Hausdorff distance. This metric measures the maximum distance for a specific percentage of data rather than the entire dataset, effectively filtering out some outlier points. The Generalized Hausdorff distance is calculated between two point clouds, and it can be applied to both the Po2Po and Po2Pl metrics. Furthermore, in [28], Javaheri et al. proposed a Point-to-Distribution (Po2D) metric. This metric is based on the Mahalanobis distance between a point in one point cloud and its K nearest neighbors in the other point cloud. They compute the mean and covariance matrix of the corresponding distribution and employ it to measure the Mahalanobis distance between points in one point cloud and their respective set of nearest neighbors in the other point cloud. These distances are then averaged to determine the final quality score. In [29], they presented a joint color and geometry point-to-distribution quality metric. This metric leverages the scale-invariance property of the Mahalanobis distance. In [30], Javaheri et al. proposed resolution-adaptive metrics. These metrics enhance the existing D1-PSNR and D2-PSNR metrics by incorporating normalization factors based on the point cloud’s rendering and intrinsic resolutions.

A feature-based point cloud quality approach computes a quality score by analyzing the differences in local and global features extracted from reference and degraded point clouds. In [31], Meynet et al. introduced the Point Cloud Multi-Scale Distortion metric (PC-MSDM). This metric serves as a measure of the geometric quality of point clouds, drawing its foundations from structural similarity principles and relying on the statistical examination of local curvature.

Viola et al. introduced a quality metric for point clouds using the histogram and correlogram of the luminance component [32]. Then, they integrated the newly proposed color quality metric with the Po2Pl MSE geometry metric (D2) using a linear model. The weighting parameter for this fusion is determined through a grid search approach.

Diniz et al. introduced the Geotex metric, a novel approach based on Local Binary Pattern (LBP) descriptors developed for point clouds, particularly focusing on the luminance component [33]. To implement this metric to point clouds, the LBP descriptor is computed within a local neighborhood corresponding to the K-nearest neighbors of each point in the other point cloud. The histograms of the extracted feature maps are generated for both the reference and degraded point clouds, and are used to calculate the final quality score employing a distance metric, such as the f-divergence [34]. In [35], Diniz et al. presented an extension of the Geotex metric. This extension incorporates various distances, with a notable focus on the Po2Pl Mean Squared Error (MSE) for assessing geometry and the distance between Local Binary Pattern (LBP) statistics [33] for evaluating color. Additionally, Diniz et al. introduced a novel quality metric in their study [36], which calculates Local Luminance Patterns (LLP) based on the K-nearest neighbors of each point in the alternative point cloud.

Meynet et al. introduced the Point Cloud Quality Metric (PCQM) [3]. It integrates geometric characteristics from a previous study [31] with five color-related features, including lightness, chroma, and hue. The PCQM is calculated as the weighted mean of the geometric and color attributes differences between the reference and degraded point clouds. In an another study, Viola et al. [7] presented the first reduced-reference quality metric, which concurrently evaluates geometry and color aspects. The authors extracted seven statistical features, including measures such as mean and standard deviation, from point clouds in reference and degraded states across various domains, including geometry, texture, and normal vectors. This process yielded a total of 21 features. The reduced quality score is calculated as the weighted average of the differences in all these features between the reference and degraded point clouds.

Inspired by the SSIM quality metric designed for 2D images, Alexiou et al. introduced a quality metric in [37] that utilizes local statistical dispersion features. These statistical characteristics are derived within a local neighborhood surrounding each point within the reference and degraded point clouds, including four distinct attributes: geometry, color, normal vectors, and curvature information. The final quality metric is derived by aggregating the differences in feature values between corresponding points in the reference and degraded point clouds. In [6], Yang et al. proposed a quality metric based on graph similarity. They identify key points by resampling the reference point cloud and construct local graphs centered at these key points for both the reference and degraded point clouds. Several local similarity features are then computed based on the graph topology, with the quality metric value corresponding to the degree of similarity between these features. Additionally, in [38], Diniz et al. extracted local descriptors that capture geometry-aware texture information from the point clouds. These descriptors include the Local Color Pattern (LCP) and various adaptations of the Local Binary Pattern (LBP) descriptor. The statistics of these descriptors are computed and used to determine the objective quality score.

A quality metric for point clouds that relies on projection involves mapping the 3D reference and degraded point clouds onto specific 2D planes. The quality score is then determined by comparing these projected images using various 2D image quality metrics. The first projection-based point cloud quality metric was introduced by Queiroz et al. in [39]. This metric begins by projecting the reference and degraded point clouds onto the six faces of a bounding cube that includes the entire point cloud. It combines the corresponding projected images and evaluates the 2D Peak Signal-to-Noise Ratio (PSNR) between the concatenated projected images from the degraded and reference point clouds. In [40], Torlig et al. introduced rendering software for visualizing point clouds on 2D screens. This software accomplishes the orthographic projection of a point cloud onto the six faces of its bounding box. Then, a 2D quality metric is applied to the projected images obtained by rendering, both for the reference and degraded point clouds. The final quality score is determined by averaging the results from the six projected image pairs. In [41], Alexiou et al. investigated how the quantity of projected 2D images impacts the correlation between subjective and objective assessments in projection-based quality metrics. The study reveals that even a single view can yield a reasonable correlation performance. Furthermore, they proposed a projection-based point cloud quality metric that assigns weights to the projected images based on user interactions during the subjective testing phase. In [42], the quality metric proposed in [40] is evaluated using different parameters, such as the number of views and pooling functions, to establish benchmarks and assess its performance under various conditions.

In [9], Liu et al. introduced a no-reference quality metric based on deep learning named the Point Cloud Quality Assessment Network (PQA-Net). This method begins by projecting the point cloud into six distinct images which undergo feature extraction through a convolutional neural network. These features are then processed by a distortion-type identification network and a quality vector prediction network to derive the final quality score. In [43], Bourbia et al. utilized a multi-view projection in 2D, which is segmented into patches, in combination with a deep convolutional neural network for evaluating the quality of point clouds.

In [44], Wu et al. introduced two objective quality metrics based on projection: a weighted view projection-based metric and a patch-projection-based metric. In both cases, 2D quality metrics are employed to assess the quality of texture and geometry maps. In particular, the patch-projection-based metric demonstrates a significant performance advantage over the weighted view projection-based metric. In [45], Liu et al. proposed a quality metric for point clouds that leverages attention mechanisms and the principle of information content-weighted pooling. Their proposed metric involves translating, rotating, scaling, and orthogonally projecting point clouds into 12 different views, and it evaluates the quality of these projected images using the IW-SSIM [46] 2D metric.

Point-based methods often prioritize geometry at the point level, neglecting color information. This limitation can be a drawback in situations where color details are significant. Focusing only on geometry may lead to an incomplete evaluation, especially when color plays a crucial role in the overall quality of the content.

The quality of feature-based methods heavily relies on the effectiveness of feature extraction techniques. Inaccurate or inadequate features can result in biased assessments.

Projection-based methods encounter the challenge of unavoidable information loss during the projection process. This loss can affect the accuracy of quality assessment, especially when critical details are compromised. Furthermore, the quality of projected images may be influenced by the angles and viewpoints employed in the projection. This sensitivity can lead to variations in the assessment results based on different projection configurations.

## 3. Proposed Method

**Figure 1:**
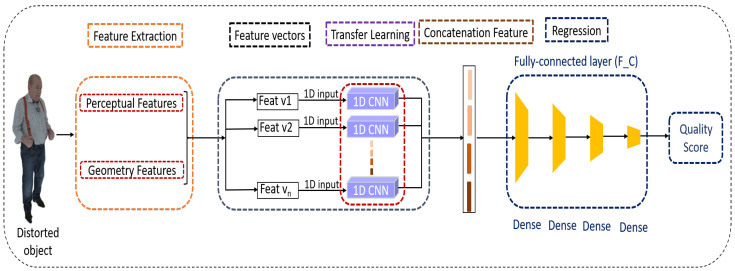
A suggested model based on transfer learning using 1D CNN.

**Figure 2:**
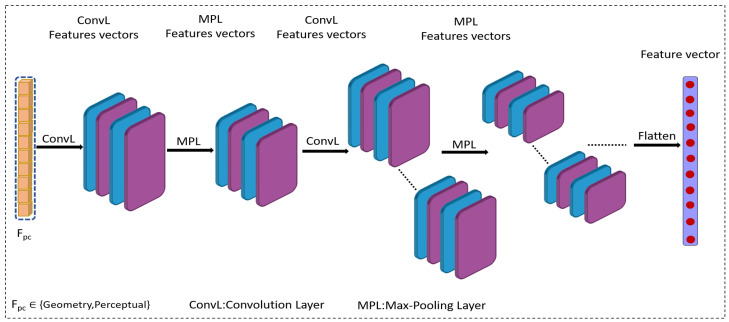
The architecture of the 1D CNN Model.

The proposed approach employs transfer learning using 1D CNN to evaluate the quality of a point cloud. Transfer learning allows leveraging the knowledge of weights and layers from a pre-existing model to speed up the learning process of a new, untrained model. We transform the 2D CNN model into a 1D CNN variant in a first time. Then, we fit the ImageNet weights of the 2D CNN model to the formed 1D CNN model. After, we use this model to produce robust features for quality regression. Finally, fully connected (F_C) layers are used as the regression model. The overall structure of the proposed method is depicted in Figure 1 and the architecture of the 1D CNN Model is illustrated in Figure 2.

### 3.1. Geometric and Perceptual Features

#### 3.1.1. Geometry-Based Features

We have selected a set of relevant geometric features to assess the quality of point clouds. These features utilize eigenvalues and eigenvectors, which are calculated for each 3D point based on its neighbors within a specified radius. The associated covariance matrix $C_{m}$, given a point Pc and its neighborhood $P_{Ngm}$, is represented by the following notation:

(1) $$C_{m}=\frac{1}{k}\sum_{i=1}^{k}{\left(P_{ci}-\bar{P_{c}})(P_{ci}-\bar{P_{c}}\right)}^{T}$$

where $P_{ci}$ and $\bar{P_{c}}$ are vectors of dimension 3 × 1, $C_{m}$ is a matrix of dimension 3 × 3, *K* denotes the size of the neighborhood $P_{Ngm}$. The eigenvector for the covariance matrix $C_{m}$ can be represented as:

(2) $$C_{m}.V_{n}=\lambda_{n}.V_{n},n\in \left\{1,2,3\right\}$$

where the relevant eigenvectors are represented by ($V_{1}$, $V_{2}$, $V_{3}$) and the eigenvalues are denoted by ($\lambda_{1}$, $\lambda_{2}$, $\lambda_{3}$), such as $\lambda_{1}$ ≥ $\lambda_{2}$ ≥ $\lambda_{3}$.

For each point cloud $PC=\left\{points\right\}$, we extract the set of geometric features defined by:

(3) $$F_{geom}=Feat_{geom}\left(pc_{i}\right)$$

where $Feat_{geom}\left(pc_{i}\right)$ signifies the geometry projection function, and $pc_{i}\in \left\{points\right\}$. The geometric feature formulations [47] are indicated as follows:

- **Linearity:** is the characteristic that denotes the degree of resemblance to a straight line:
  (4) $$Lin=\frac{\lambda_{1}-\lambda_{2}}{\lambda_{1}}$$
- **Planarity:** is employed to assess the resemblance or similarity to a planar surface:
  (5) $$Plan=\frac{\lambda_{2}-\lambda_{3}}{\lambda_{1}}$$
- **Anisotropy:** is employed to demonstrate discrepancies in geometrical characteristics across various directions:
  (6) $$Anis=\frac{\lambda_{1}-\lambda_{3}}{\lambda_{1}}$$
- **Sphericity:** is the metric used to quantify the degree of resemblance between the shape of an object and that of a perfect sphere:
  (7) $$Sph=\frac{\lambda_{3}}{\lambda_{1}}$$
- **Omnivariance:** is a geometric descriptor used to quantify point cloud data’s overall variability or diversity in three-dimensional space. It captures the dispersion of points in all directions and provides a measure of the spatial distribution of the points:
  (8) $$Omni={\left(\lambda_{1}*\lambda_{2}*\lambda_{3}\right)}^{\frac{1}{3}}$$
- **Eigenentropy:** is a mathematical measure that quantifies the level of disorder or randomness in a dataset, particularly in the context of analyzing 3D point clouds or spatial distributions. It assesses how dispersed or organized the data points are within a given neighborhood or region:
  (9) $$Eigen=-\sum_{i=1}^{3}\lambda_{i}\ln\left(\lambda_{i}\right)$$
- **Sphere-fit:** is often employed in various applications such as computer graphics, computer-aided design (CAD), and computer vision, where finding an accurate and robust approximation of a sphere to a set of scattered points is essential. It plays a significant role in many fields involving 3D point cloud data analysis and manipulation.

#### 3.1.2. Perceptual-Based Features

For each point cloud, we extract the set of perceptual features $F_{perc}$ defined by:

(10) $$F_{perc}=Feat_{perc}\left(pc_{i}\right)$$

where $Feat_{perc}\left(pc_{i}\right)$ indicates the perceptual projection function. We have chosen color, curvature (Curv), and saliency (Sal) for perceptual features.

- **Color:** This plays a crucial role in evaluating visual quality. In a colored point cloud, each point’s color is directly derived from its color information. Typically, the color information in 3D models is stored using RGB channels. However, the RGB color space has shown limited correlation with human perception. We choose to use the LAB color transformation for color feature projection as a solution. This method has been widely embraced for numerous quality assessment applications [48].
- **Saliency:** This is a crucial aspect within the human visual system, involving allocating human attention or eye movements in a given scene. Identifying these remarkably perceptible areas is important in computer vision and computer graphics.
- **Curvature:** This refers to the amount by which a curve, surface, or object deviates from being perfectly straight or flat at a specific point:
  (11) $$Curv=\frac{\lambda_{3}}{\lambda_{1}+\lambda_{2}+\lambda_{3}}$$

### 3.2. Feature Encoder

We convert the 2D CNN architecture into a 1D CNN version and adjust the weights derived from ImageNet for the 1D CNN model. Then, we produce the features through the following process:

(12) $$F_{i}=1D\_CNN\left(F_{pc}\right)$$

where $F_{pc}\in \left\{F_{geom},F_{perc}\right\}$, $F_{i}$ represents the produced features for each $F_{pc}$, while 1DCNN(.) refers to the module responsible for robust features production using the 1D CNN architecture.

### 3.3. Convert the 2D CNN Model to a 1D CNN Model

Deep 2D CNNs have played a crucial role in computer vision tasks and have achieved remarkable success in various applications, including image recognition, object detection, facial recognition, and more. The pre-trained models (e.g., VGG, ResNet, MobileNet) have been used to tackle specific tasks, depending on the dataset and problem.

Leveraging the 2D CNN pre-trained model enables the acceleration of deep model training for other tasks through transfer learning [49,50]. This advantage lies in its ability to achieve satisfactory results without requiring large amounts of labeled data or extensive computational resources.

Converting a 2D CNN model to a 1D CNN model typically involves modifying the fully connected layers of the model to handle 1D data instead of 2D data. The original 2D CNN architecture processes 2D images, where each image has height, width, and color channels (e.g., RGB). In contrast, a 1D model processes sequential data, such as text or time series data, with only one dimension. To convert 2D CNN to 1D CNN, we need to adapt the fully connected layers to work with the 1D input. Here is an overview of the followed steps:

1. Remove the last few layers: In a 2D CNN model, the final layers are usually fully connected layers responsible for image classification. We need to remove these layers since they are designed for 2D data.
2. Flatten the output: Since 1D data have only one dimension, we need to flatten the output of the last convolutional layer to convert it into a 1D format.
3. Add new fully connected layers: After flattening, we add new fully connected layers designed to handle 1D data. These layers should have an appropriate number of neurons and activations suitable for the specific task you want to solve.
4. Adjust input data: The input data fed into the model should also be converted to 1D format to match the new architecture.
5. Adjust Output Layer: Finally, the output layer of the 2D model may need to be modified to match the desired output for the 1D model. For example, for regression tasks, it may need to output a single value, while for classification tasks, it may need to produce class probabilities.
6. Transfer Weights: Once the architecture is adjusted, the weights of the 2D model can be transferred to the corresponding layers in the 1D model. However, since some layers may have been removed or modified, care must be taken to ensure the weights are transferred appropriately.

It is important to note that converting a model from 2D to 1D is not always straightforward, especially if the model was initially designed for 2D image. The success of such a conversion heavily depends on the nature of the problem you want to solve with the 1D model.

### 3.4. Quality Prediction

The feature extraction step permits us to obtain a feature vector that captures the distinctive attributes of the 3D model. For our experiment, we suggest utilizing a four-layer fully connected (F_c), the associated hyperparameters are presented in Table 1. We integrate the aforementioned features to make predictions about perceptual quality. Finally, the estimated quality scores Q_s can be calculated as follows:

(13) Q_s=F_c(F)

We minimize the regression loss during each training batch using the Mean Squared Error (MSE). This later serves the purpose of maintaining the proximity of predicted values to the quality labels, and this relationship can be expressed as follows:

(14) $$Loss_{MSE}=\frac{1}{n}\sum_{i=1}^{n}{\left(Qs_{i}-MOS_{i}\right)}^{2}$$

where *n* represents the number of distortions present in a given database. The database provides Mean Opinion Scores ($MOS_{i}$) that define the subjective quality assessment, while $Qs_{i}$ represents the objective quality score obtained through a specific method.

## 4. Experimental Setup

### 4.1. Databases

In our study, we use three of the most popular in the field of point cloud quality assessment that are: the subjective point cloud assessment database (SJTU-PCQA) [51], the Waterloo point cloud assessment database (WPC) [45], and the ICIP2020 point cloud assessment database (ICIP2020) introduced in [52].

- The SJTU-PCQA database [51] contains 420 point cloud samples derived from 10 reference point clouds. Each reference point cloud undergoes seven common types of distortions at six different levels. More precisely, the distortions are acquired using compression based on Octree (OT), noise in color (CN), noise in Gaussian geometry (GGN), Downscaling (DS), Downscaling and Color noise (D + C), Downscaling and Geometry Gaussian noise (DCG), and noise in color combined with Gaussian geometry (C + G). However, only 9 reference point clouds and their corresponding distorted samples are publicly available, resulting in 378 (9 × 6 × 7) point cloud samples for our experiment. Mean Opinion Scores (MOSs) are provided within the range of [0,1].
- The WPC dataset [45] comprises 20 original reference point clouds and 740 altered point clouds created from these references using five distinct forms of distortion. These distortions encompass Downsampling (DS), contamination by Gaussian noise (GN), G-PCC (Trisoup), GPCC (Octree), and V-PCC.
- The ICIP2020 database [52] comprises six reference point clouds that incorporate both texture and geometry information. Additionally, it includes 90 distorted versions obtained using 3 compression methods: G-PCC Octree, G-PCC Trisoup, and VPCC, each at 5 different quality levels ranging from low to high.

**Figure 3:**
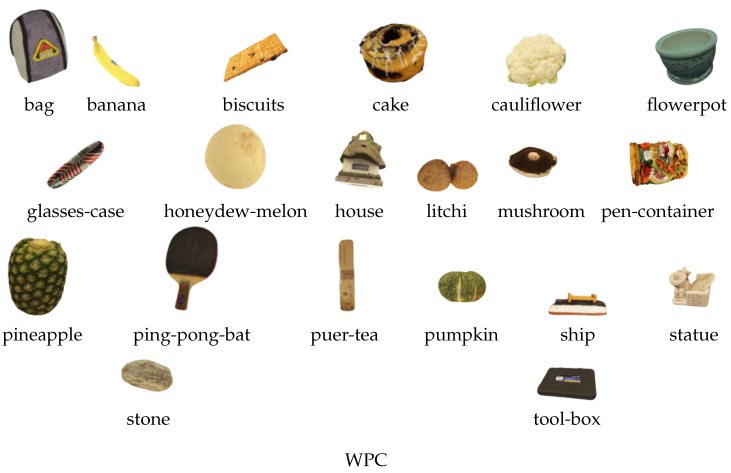

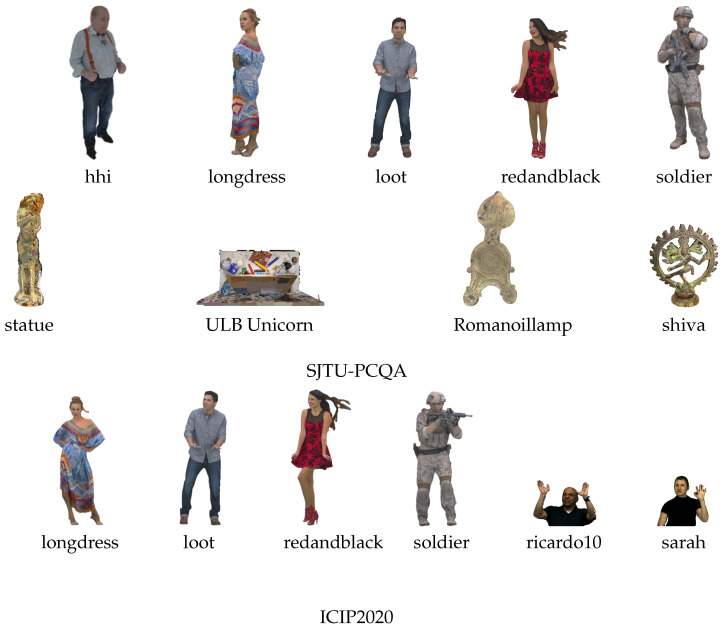
Displays the reference samples sourced from the WPC, SJTU-PCQA, and ICIP2020 databases [45,51,52].

Figure 3 displays the reference samples of the WPC, SJTU-PCQA, and ICIP2020 databases.

### 4.2. Implementation Parameters

To assess the proposed approach against other learning-based NR-PCQA metrics, we partitioned ICIP2020, SJTU-PCQA, and WPC datasets into training and testing sets. The division of reference point clouds for these three datasets ensured that 80% of the samples were allocated for training, leaving 20% for testing purposes. The Adam optimizer is utilized throughout the training phase, initializing with a learning rate of 1 × 10^−4^, while maintaining a batch size of 10. Furthermore, the model is trained over 50 epochs. We performed the test using a computer equipped with an Intel (R) Core (TM) i7-11800H @ 2.30 GHz, 32 GB of RAM, and an NVIDIA GeForce RTX 3060 Laptop GPU on the Windows platform.

### 4.3. Evaluation Metrics

Four evaluation criteria are employed to find the relation between the predicted scores and MOSs. These criteria are the Spearman Rank Correlation Coefficient (SRCC), Kendall’s Rank Correlation Coefficient (KRCC), Pearson Linear Correlation Coefficient (PLCC), and Root Mean Squared Error (RMSE). These criteria are defined as follows:

(15) $$PLCC=\frac{\sum_{j=1}^{n_{j}}\left(Qs_{j}-\bar{Qs})(MOS_{j}-\bar{MOS}\right)}{\sqrt{\sum_{j=1}^{n_{j}}{\left(Qs_{j}-\bar{Qs}{)}^{2}(MOS_{j}-\bar{MOS}\right)}^{2}}}$$

(16) $$SRCC=1-\frac{6\sum_{j=1}^{n_{j}}{\left(rank\left(MOS_{j}\right)-rank\left(Qs_{j}\right)\right)}^{2}}{n_{j}\left(n_{j}^{2}-1\right)}$$

(17) $$KRCC=\frac{n_{cj}-n_{dj}}{\frac{1}{2}\left(n_{j}^{2}-n_{j}\right)}$$

(18) $$RMSE=\sqrt{\frac{1}{n_{j}}\sum_{i=1}^{n_{j}}{\left(Qs_{j}-MOS_{j}\right)}^{2}}$$

where $n_{j}$ represents the number of distortions present in a given database and $n_{cj}$ and $n_{dj}$ indicate the total number of consistent and inconsistent in the database. The dataset provides Mean Opinion Scores ($MOS_{j}$) that define the subjective quality assessment, while $Qs_{j}$ represents the objective quality score obtained through a specific method.

## 5. Experimental Results

This section deals with our experimental results, including the performance analysis of the studied networks, the ablation study, results achieved through comparisons with state-of-the-art methods, and cross-database evaluations.

### 5.1. Network Performance

Convolutional Neural Networks (CNNs) come in various architectures and configurations, each designed for specific tasks and use cases. To explore how CNNs impact performance quality, we execute experiments using six distinct CNN architectures pre-trained on the ImageNet dataset: MobileNet, ResNet, DenseNet, ResNeXt, SE-ResNet, and VGG.

- **ResNet [53]:** Kaiming et al. introduced the Residual Neural Network (ResNet). This network was designed to simplify the training process of deep networks by expediting training speeds. Various iterations of ResNet, such as ResNet 18, ResNet 34, ResNet 50, and ResNet 101, among others, have been suggested.
- **ResNeXt [54]:** ResNeXt is a convolutional neural network architecture aiming to improve deep learning models’ efficiency and performance. ResNeXt builds upon the Residual Network (ResNet) architecture by introducing “cardinality”.
- **MobileNet [55]:** MobileNet is a family of neural network architectures designed explicitly for efficient inference on mobile and embedded devices. MobileNets are known for their lightweight and computationally efficient nature while maintaining reasonable accuracy on various tasks, especially in computer vision.
- **DenseNet [56]:** DenseNet, short for Densely Connected Convolutional Network, is a neural network architecture proposed by Huang et al. DenseNet introduces a unique connectivity pattern among layers, aiming to address some limitations of traditional neural network architectures, such as vanishing gradients, feature reusability, and ease of training deeper networks.
- **SEResNet [57]:** SEResNet (Squeeze-and-Excitation ResNet) is an extension of the ResNet (Residual Network) architecture that incorporates a mechanism called “Squeeze-and-Excitation” to enhance feature learning and representation.
- **VGG [58]:** The VGG network, a deep Convolutional Neural Network (CNN) demonstrated notable success in the ILSVRC 2014 competition. Diverse iterations of VGG, featuring distinct convolutional layer configurations have been created, including VGG11, VGG13, VGG16, and VGG19.

The characteristics of these networks are illustrated in Table 1 and their corresponding results can be found in Table 2 and Table 3.

We evaluate the impact of network architecture and depth on the proposed method’s performance. Residual Networks (ResNets) demonstrate progressive improvement with increasing depth, with ResNet34 achieving better inter-database correlation than ResNet18. However, ResNeXt50 exhibits superior performance on SJTU and ICIP2020 datasets, but struggles on the WPC dataset. MobileNetV2 stands out for its consistent performance across all datasets despite having fewer parameters and lower memory footprint. DenseNet201 suffers from performance degradation and higher Root Mean Squared Error (RMSE) across databases. SE-ResNet50 emerges as the top performer with exceptional metrics such as Pearson Correlation Coefficient (PLCC), Spearman Rank Correlation Coefficient (SRCC), Kendall Rank Correlation Coefficient (KRCC), and consistently low RMSE across all datasets. Surprisingly, VGG16 and VGG19, known for strong performance, excel on all metrics, demonstrating remarkable generalizability, particularly on SJTU and ICIP2020 datasets.

Results obtained from Table 1, Table 2 and Table 3 reveal a classic complexity-accuracy trade-off within the evaluated DCNN architectures. While deeper models such as VGG-16 and VGG-19 achieve higher accuracy, they exhibit significantly larger numbers of parameters and require more computational resources for inference compared to lightweight models such as MobileNetV2. ResNet-101, SEResNet-50, ResNeXt-50, and DenseNet-201 offer a potential middle ground, balancing accuracy with computational efficiency. However, these models come at the cost of increased memory footprint and processing power compared to shallower architectures such as ResNet-18 or ResNet-34. Our evaluation of the impact of varying depth and parameter counts across MobileNet, ResNet, DenseNet, and VGG models suggests that these factors may not significantly influence performance outcomes. This finding warrants further investigation to determine the optimal architecture for specific tasks considering the application’s resource constraints and target accuracy requirements.

**Figure 4:**
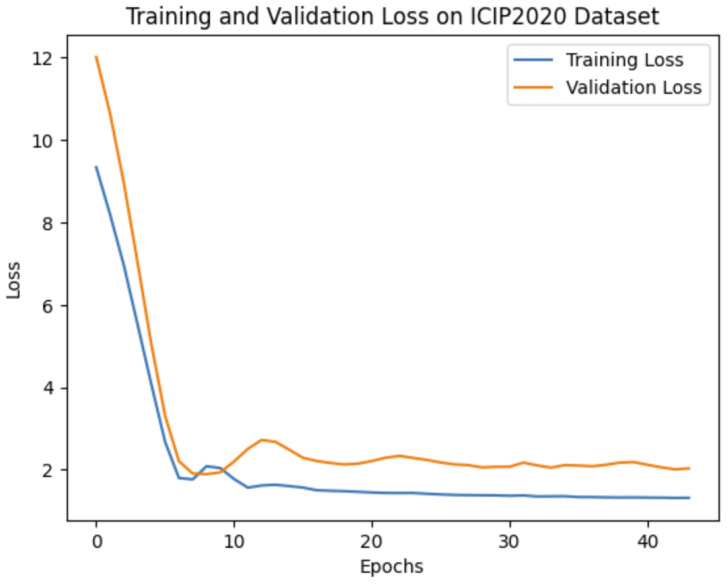
Training and validation loss of 1D CNN VGG16 on the ICIP2020 dataset.

**Figure 5:**
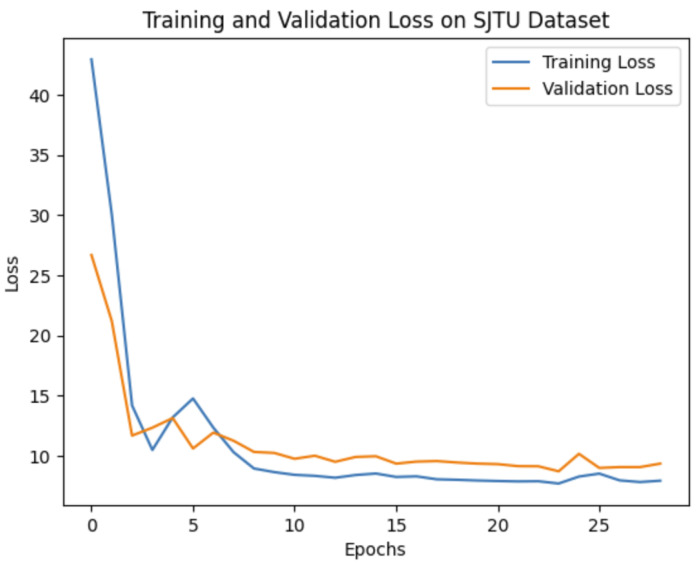
Training and validation loss of 1D CNN VGG16 on the SJTU dataset.

**Figure 6:**
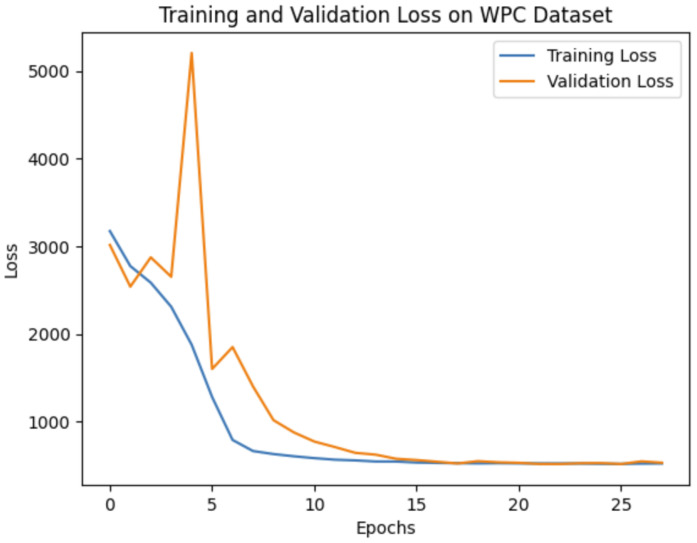
Training and validation loss of 1D CNN VGG16 on the WPC dataset.

Our experiments demonstrate that the choice of network architecture significantly impacts model performance, even more so than increasing the depth or number of parameters in the model. When selecting a model, it is crucial to consider the application’s specific requirements. If prioritizing real-time performance or running on mobile devices is essential, then a lightweight model like MobileNetV2 might be preferable. On the other hand, if achieving the highest possible accuracy is the primary goal, then deeper models such as VGG16 or SE-ResNet50 are more suitable options. To evaluate our method’s effectiveness against leading benchmarks, we opted to leverage the pre-trained VGG16 convolutional neural network (CNN). The training and validation curves for VGG16 on all three databases are shown in Figure 4, Figure 5 and Figure 6.

**Figure 7:**
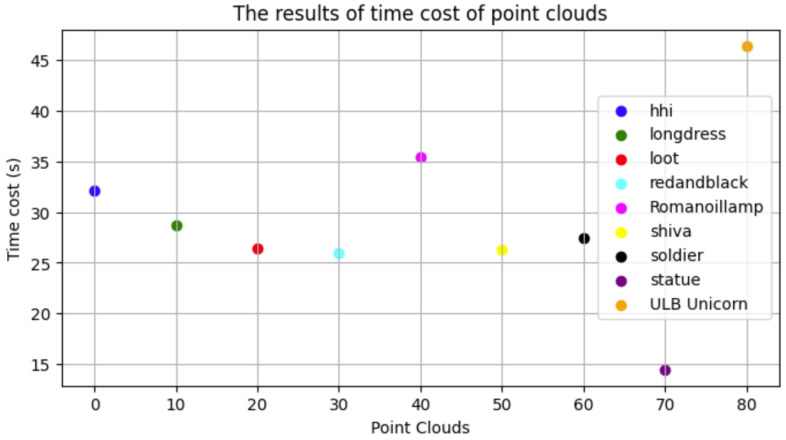
The results of the time cost of point clouds using 1D CNN VGG16 on the SJTU dataset.

Since the suggested approach functions directly from the 3D model, we also pay attention to computational efficiency. The results of execution time on the SJTU-PCQA database are presented in Figure 7. The proposed method has an average time cost of 29.27 s, compared to approximately 39.14 s for PCQM. Although the FR method for PCQM needs to load both deformed and reference point cloud models simultaneously, while the proposed NR method only needs to load the deformed point cloud model, our method demonstrates a lower average time cost. This suggests that our approach achieves relatively significant computational efficiency.

### 5.2. Ablation Study

To assess the effectiveness and contributions of various types of features perceptual and geometry, we conducted individual performance tests for each feature. This allowed us to analyze the contributions of features by assessing their performance within various combinations. Table 4, Table 5 and Table 6 present the performance outcomes of the ablation study. Where ‘Anis’, ‘Lin’, ‘Plan’, ‘Sph’, ‘Omni’, ‘Sph_fit’, ‘Eigen’, ‘Curv’, ‘Sal’, ‘All_geom’, and ‘All_perceptual’ correspond to Anisotropy, Linearity, Planarity, Sphericity, Omnivariance, Sphere-fit, Eigenentropy, Curvature, Saliency, all geometry features, and all perceptual features, respectively. Additionally, ‘L’, ‘A’, and ‘B’ represent the luminance and chrominance channels within the LAB color space.

Results obtained on ICIP2020 dataset (Table 4) show distinct performances among various features (e.g., ‘Anis’, ‘Lin’, ‘Plan’). ‘Anis’ and ‘Sph’ exhibit strong individual performance, while ‘Lin’, ‘Plan’, and ‘Omni’ are less effective. However, progressively integrating these features with ‘Anis’ significantly improves all evaluation metrics. The ‘All_geom’ feature set, combining all geometric features, achieves superior performance throughout, highlighting the benefit of this combination.

For perceptual features, ‘Curv’ is the best performer, while ‘L’, ‘A’, ‘B’, and ‘Sal’ have less individual impact. Merging ‘L’, ‘A’, and ‘B’ followed by ’Curv’ integration results in substantial gains, emphasizing the importance of their collective influence. The ‘All_perceptual’ set, regrouping all perceptual features, demonstrates significant performance, suggesting a complementary interaction between these features.

Finally, integrating geometric and perceptual features, the ‘All’ combination outperforms all individual and grouped sets across all metrics. This comprehensive set not only achieves optimal performance but also underscores the efficacy of combining complementary features for enhanced point cloud quality assessment.

Analyzing the SJTU dataset (Table 5) reveals interesting insights into feature performance. Among geometric features, ‘Plan’ exhibits the strongest overall performance across all metrics (PLCC, SRCC, KRCC). ‘Sph’ follows closely, particularly excelling in PLCC and SRCC compared to ‘Plan’. While ‘Anis’ demonstrates decent performance, particularly in SRCC, it falls behind ‘Plan’ and ‘Sph’. ‘Omni’ achieves the highest PLCC but struggles with SRCC and KRCC compared to ‘Sph’. ‘Sph_fit’ stands out with significant improvements across all metrics, surpassing other geometric features in a performance jump. However, ‘Engein’ performs similarly to the moderate ‘Anis’. Combining all geometric features in ‘All_geom’ significantly improves individual or partial combinations, highlighting the benefit of feature fusion.

For perceptual features, ‘L’, ‘A’, and ‘B’ exhibit moderate performance, with ‘B’ slightly edging out the others. ‘Curv’ demonstrates improvement over this group, while ‘Sal’ substantially gains performance. As with geometric features, combining these perceptual features into ‘All_perceptual’ leads to further metric improvements.

Finally, the ‘All’ combination, integrating geometric and perceptual features, outperforms all individual and partial combinations across all metrics. This comprehensive feature set stresses the importance of incorporating diverse feature information for optimal point cloud quality assessment. The observed incremental improvements with feature additions, the impact of combining features on metrics, and the context-dependent selection of features all emphasize the effectiveness of a holistic approach in this task.

The scores reported on WPC dataset (Table 6) reveal distinct characteristics for each geometric feature (‘Anis’, ‘Lin’, ‘Plan’, ‘Sph’, ‘Omni’, ‘Sph_fit’, ‘Engein’). ‘Plan’ demonstrates superior performance with higher correlations (PLCC: 0.29) and lower error (RMSE: 22.57) compared to ‘Sph’ (PLCC: 0.10, RMSE: 23.45). However, feature fusion leads to significant improvements. Combining ‘Anis’ + ‘Lin’ + ‘Plan’ elevates PLCC to 0.41 and reduces RMSE to 21.57. The ‘All_geom’ feature set, which combines all the geometric features, greatly improves (PLCC: 0.67, RMSE: 17.42). This means that using all these features together works much better than using them alone. Similar observations hold for perceptual features (‘L’, ‘A’, ‘B’, ‘Curv’, ‘Sal’). While individual features exhibit varying performance impacts, their combination (‘L’ + ‘A’ + ‘B’ + ‘Curv’) leads to improved metrics compared to their contributions. Furthermore, the ‘All_perceptual’ set, regrouping all perceptual features, demonstrates significant gains in correlations (PLCC: 0.61) and error reduction (RMSE: 21.43).

The ‘All’ combination, integrating geometric and perceptual features, achieves the peak performance across all metrics. This comprehensive set boasts strong correlations (PLCC: 0.93) and minimal error (RMSE: 8.55). This analysis highlights the cumulative improvements observed as more features are incorporated, with the ‘All’ set demonstrably superior for point cloud quality assessment. This underlines the critical role of geometric and perceptual information in achieving optimal quality evaluation.

Across the three evaluated datasets, the results indicate that geometry features play a more significant role in determining the final quality score. This observation could be attributed to the fact that three databases contain a greater variety of geometry distortions than perceptual distortions, and human perception of point clouds tends to place a higher emphasis on geometry-related information.

### 5.3. Performance Comparison with the State-of-the-Art

In this section, we perform a comparative analysis of our proposed method against current benchmarks, including FR-PCQA (PSNR [5], SSIM [5], PB-PCQA [51], M-p2po [24], M-p2pl [4], H-p2po [24], H-p2pl [4], PSNRYUV [40], PCQM [3], GraphSIM [6], PointSSIM [37], TCDM [59], and MMD [28]), RR-PCQA (PCMRR [7]), and NR-PCQA (BRISQUE [11], PQA-Net [9], NIQE [12], ResSCNN [10], MVP-PCQA [43], MM-PCQA [60], and 3D-NSS [13]).

The experimental outcomes of PCQA using the SJTU-PCQA, WPC, and ICIP2020 databases are presented in Table 7, Table 8 and Table 9. The top-performing results in each column are highlighted in bold. Across all three databases, it is evident that the FR-PCQA methods (PSNR [5], M-p2po [24], M-p2pl [4], H-p2po [24], and H-p2pl [4]) demonstrate comparatively lower performance. This can be attributed to their reliance solely on geometric structure without incorporating color information. In contrast, superior performance is observed in metrics such as MMD [28], PSNRYUV [40], PCQM [3], GraphSIM [6], PointSSIM [37], and TCDM [59], which includes color information for assessing point clouds. However, it is important to note that evaluating these methods relies on reference information, a component often unavailable in practical applications. Regarding RR methods, The PCMRR metric produces poor results for all correlation metrics. This might be explained by the extensive use of features within their method, which could make it less generalized to different types of degradation.

For NR methods, one can see that our method presents the most superior performance across all three databases, outperforming the compared NR-PCQA methods by a significant margin. For example, our approach outperforms the NR-PCQA method in second place by approximately 0.04, 0.04 (PLCC, SRCC) for MM-PCQA on the SJTU-PCQA database, and by 0.08, 0.07 (PLCC, SRCC) for MVP-PCQA on the WPC database. There are significant performance drops from the SJTU-PCQA and ICIP2020 databases to the WPC database because the latter contains more complex distortion parameters, which are more difficult for PCQA models. Moreover, within the SJTUPCQA database, distorted point clouds contain mixed distortions, while the WPC database introduces a single type of distortion to individual point clouds. Point clouds with mixed distortions appear to exhibit greater quality distinguishability when subjected to similar distortion levels. Furthermore, the WPC database contains twice the number of reference point clouds compared to the SJTU-PCQA database. Our approach exhibits a relatively small drop in performance compared to most other methods. For instance, when moving from the SJTU-PCQA database to the WPC database, our method demonstrates a decrease of 0.03 in PLCC and 0.02 in SRCC values. Top-performing PCQA methods, except for PQA-Net, exhibit a larger performance decline of 0.15 and 0.14 in both PLCC and SRCC values, respectively. Therefore, it is clear that our approach is more robust to more complex distortions.

The overall effectiveness may not accurately reflect the performance for specific distortion types. Consequently, we assess how FR, RR, and NR metrics perform in the face of various point cloud distortions across the three databases. Evaluation measures such as PLCC and SRCC scores are presented in Table 10, Table 11 and Table 12. The top performance for each distortion type within each database is highlighted in bold, indicating the best results among all competing metrics.

On the ICIP2020 database (Table 10), our method surpasses all the compared methods across various distortion types (VPCC, G-PCC Trisoup, and G-PCC Octree), demonstrating superior performance across the entire database.

Within the SJTU-PCQA database (Table 11), our method shows the strongest correlation coefficient outcomes across all distortions, outperforming the state-of-the-art metrics in both PLCC and SRCC correlation coefficients. It is important to highlight that the correlation values of our method and all other state-of-the-art methods are lower in the SJTU-PCQA dataset compared to the ICIP2020 dataset. This difference could be explained by the various types of degradation present in the two databases. While the ICIP2020 database mostly features compression-related distortions, the SJTU database presents more difficult degradation types such as acquisition noise, resampling, and their combinations (Octree-based compression (OT), Color noise (CN), Geometry Gaussian noise (GGN), Downsampling (DS), Downscaling and Color noise (D + C), Downscaling and Geometry Gaussian noise (D + G), and Color noise and Geometry Gaussian noise (C + G)). We conduct a comparison of PLCC and SRCC values for each of the seven degradation types. As depicted in Table 11, our model demonstrates robust performance across all degradation types, exhibiting strong correlations with the subjective quality scores.

In the WPC database (Table 12), our method shows top correlation coefficient results across all distortions, outperforming all the methods compared throughout the entire database. It is important to highlight that our model performs better even on the larger and more complex databases, demonstrating its remarkable robustness.

Based on these results, it can be concluded that our proposed model ranks first among NR methods on SJTU-PCQA, WPC, and ICIP2020 databases. Additionally, our model achieves satisfactory results compared to state-of-the-art FR 3D-QA metrics. A notable advantage of our method is that it does not require original point clouds for reference, demonstrating its ability to extract quality-aware features and provide relatively accurate quality levels for colored point clouds.

### 5.4. Cross-Database Evaluation

A cross-database evaluation was performed to assess the generalization capability of the proposed method, and the experimental results are displayed in Table 13. Considering the size of the WPC database (740 samples), our primary focus was training the models using this database and conducting the test on the SJTU PCQA dataset (378 samples). Among the comparison models, 3D-NSS [13] demonstrates the lowest PLCC and SRCC values at 0.2344 and 0.1817, respectively. PQA-net [9] follows with improved scores of 0.6102 (PLCC) and 0.5411 (SRCC), displaying improved performance. MM-PCQA [60] further elevates the evaluation metrics, achieving 0.7779 (PLCC) and 0.7693 (SRCC). However, the best performance results come from the proposed model, with higher PLCC (0.8119) and SRCC (0.8193) values, surpassing all other models assessed in this study. These results suggest that the proposed model exhibits a higher correlation with ground truth data for evaluating point cloud quality, indicating its potential for more accurate quality evaluations compared to existing NR-PCQA methods such as 3D-NSS, PQA-net, and MM-PCQA within this context.

## 6. Conclusions

In this paper, we have introduced a novel methodology for assessing the quality of 3D point clouds using a one-dimensional model based on the Convolutional Neural Network (1D CNN). Through extensive experiments and evaluations, we have demonstrated the effectiveness of our approach in predicting subjective point cloud quality under various distortions. Our model consistently outperformed all competing methods by leveraging transfer learning and focusing on geometric and perceptual features.

The results of our evaluations across different distortion types and databases provide valuable insights into the performance of the proposed method. Our model achieves robust performance across all distortion types within each database in the ICIP2020 and WPC databases by recording top correlation coefficient results across all distortions.

The success of our approach can be attributed to its ability to effectively capture and analyze geometric and perceptual features in 3D point clouds, enabling accurate quality assessment without the need for reference information. The model’s generalization capability, as demonstrated in cross-database evaluations, further highlights its potential for real-world applications.

In conclusion, the proposed method is a promising solution for automated point cloud quality assessment, offering enhanced accuracy and reliability compared to existing techniques. By combining advanced deep learning strategies with transfer learning, our approach advances the field of point cloud quality assessment and opens up new possibilities for improving visual quality evaluation in diverse domains.

## Figures and Tables

**Table 1:** The characteristics of the employed networks.

Model	Parameters	GPU Memory Management	Performance
ResNet18	∼11.2 M	Moderate	Balanced efficiency and accuracy
ResNet34	∼21.3 M	Moderate	Improved over ResNet18
ResNet101	∼44.6 M	Moderate to High	Increased capacity
ResNeXt50	∼25 M	Moderate	Enhanced capacity
MobileNetV2	∼3.4 M	Low to Moderate	Efficient, lower memory footprint
DenseNet201	∼20 M	Moderate	Dense connectivity, good accuracy
SE-ResNet50	∼28.1 M	Moderate to High	Attention mechanisms, good performance
VGG16	∼138 M	High	Heavy memory usage, high accuracy
VGG19	∼144 M	High	Similar to VGG16, increased parameters

**Table 2:** Performance of different architectures on the ICIP2020. The best two performances for each database is emphasized in bold.

Model	ICIP2020			
	PLCC	SRCC	KRCC	RMSE
ResNet18	0.9117	0.9319	0.7862	0.5076
ResNet34	0.9244	0.8976	0.7407	0.5164
ResNet101	0.8542	0.9179	0.7619	0.6587
ResNeXt50	0.9066	0.8540	0.6666	0.5757
MobileNetV2	0.9000	0.8908	0.7407	0.6223
DenseNet201	0.8515	0.8638	0.6984	0.7232
SE-ResNet50	**0.9334**	**0.9486**	**0.8441**	**0.4503**
VGG16	**0.9678**	**0.9699**	**0.9100**	**0.2597**
VGG19	0.8900	0.8815	0.7018	0.6129

**Table 3:** Performance of different architectures on the SJTU-PCQA and WPC. The best two performances for each database is emphasized in bold.

Model	SJTU-PCQA				WPC			
	PLCC	SRCC	KRCC	RMSE	PLCC	SRCC	KRCC	RMSE
ResNet18	0.8373	0.8314	0.6481	1.3186	0.8112	0.8021	0.6241	14.4248
ResNet34	0.8921	0.8534	0.6853	1.0655	0.8496	0.8502	0.6889	11.3027
ResNet101	0.8315	0.8340	0.6502	1.3478	0.8133	0.8165	0.6409	12.4597
ResNeXt50	0.8655	0.8588	0.7102	1.2176	0.7677	0.7524	0.5601	15.3643
MobileNetV2	0.8843	0.8823	0.7015	1.1130	0.8654	0.8668	0.6808	11.9486
DenseNet201	0.7620	0.7552	0.5723	1.5733	0.8061	0.8044	0.6260	14.1506
SE-ResNet50	**0.8987**	**0.8916**	**0.7647**	**1.0512**	**0.8902**	**0.8866**	**0.8082**	**10.7401**
VGG16	**0.9676**	**0.9576**	**0.8454**	**0.6133**	**0.9062**	**0.9056**	**0.7554**	**10.2492**
VGG19	0.8307	0.8017	0.6151	1.3803	0.8273	0.8260	0.6510	13.6159

**Table 4:** Results of the ablation study’s performance on ICIP2020. The optimum performance for each database is highlighted in bold.

Type	Feature	ICIP2020			
		PLCC	SRCC	KRCC	RMSE
Geometry	Anis	0.6055	0.6817	0.4973	1.2251
	Lin	0.5172	0.2731	0.2328	1.4148
	Plan	0.3999	0.2039	0.0846	1.4498
	Sph	0.6447	0.6440	0.4550	1.0988
	Omni	0.5160	0.4454	0.2857	1.3031
	Sph_fit	0.5801	0.6764	0.4973	1.4379
	Eigen	0.2322	0.2769	0.2116	1.3650
	Anis + Lin	0.6777	0.6200	0.4338	1.0993
	Anis + Lin + Plan	0.8310	0.8111	0.6243	0.9880
	Anis + Lin + Plan + Sph	0.8384	0.8058	0.6455	0.7350
	Anis + Lin + Plan + Sph + Omni	0.8708	0.8231	0.6666	0.6675
	Anis + Lin + Plan + Sph + Omni + Sph_fit	0.8881	0.8480	0.6772	0.6303
	All_geom	0.9049	0.8924	0.7513	0.5917
Perceptual	L	0.1984	0.3167	0.2010	1.3735
	A	0.2563	0.1918	0.1693	1.3519
	B	0.2042	0.1813	0.1375	1.5143
	Curv	0.3705	0.3950	0.3174	1.4244
	sal	0.2977	0.2129	0.1587	1.2319
	L+A+B	0.8555	0.8465	0.6349	0.7312
	L + A + B + Curv	0.8715	0.8532	0.6560	0.6912
	All_perceptual	0.8961	0.8525	0.6455	0.6316
ALL	**All**	**0.9678**	**0.9699**	**0.9100**	**0.2597**

**Table 5:** Results of the ablation study’s performance on SJTU. The optimum performance for each database is highlighted in bold.

Type	Feature	SJTU-PCQA			
		PLCC	SRCC	KRCC	RMSE
Geometry	Anis	0.1747	0.2954	0.1944	2.4061
	Lin	0.1460	0.1521	0.1173	2.3887
	Plan	0.2180	0.2372	0.1551	2.3924
	Sph	0.2306	0.3289	0.2169	2.3885
	Omni	0.3828	0.1627	0.1141	2.2534
	Sph_fit	0.5768	0.3988	0.2649	2.3809
	Eigen	0.2107	0.2245	0.1459	2.3803
	Anis + Lin	0.2414	0.2366	0.1769	2.3939
	Anis + Lin + Plan	0.4380	0.4169	0.2836	2.2645
	Anis + Lin + Plan + Sph	0.5864	0.5656	0.4008	2.1564
	Anis + Lin + Plan + Sph + Omni	0.6553	0.6285	0.4628	1.8705
	Anis + Lin + Plan + Sph + Omni + Sph_fit	0.6598	0.6564	0.4892	1.8356
	All_geom	0.7489	0.7185	0.5147	1.6824
Perceptual	L	0.1771	0.1630	0.1049	2.3678
	A	0.1934	0.1623	0.1164	2.3939
	B	0.2450	0.2594	0.1713	2.3831
	Curv	0.1664	0.2192	0.1316	2.4045
	sal	0.3253	0.3840	0.2808	2.4053
	L + A + B	0.3572	0.3414	0.2382	2.2578
	L + A + B + Curv	0.4484	0.4325	0.2972	2.2555
	All_perceptual	0.6059	0.6351	0.4600	2.0780
ALL	**All**	**0.9676**	**0.9576**	**0.8454**	**0.6133**

**Table 6:** Results of the ablation study’s performance on WPC. The optimum performance for each database is highlighted in bold.

Type	Feature	WPC			
		PLCC	SRCC	KRCC	RMSE
Geometry	Anis	0.1914	0.1712	0.1205	23.4895
	Lin	0.2183	0.1266	0.0834	23.0657
	Plan	0.2904	0.2222	0.1620	22.5680
	Sph	0.1015	0.1026	0.0637	23.4544
	Omni	0.1377	0.1566	0.0981	23.3771
	Sph_fit	0.2701	0.1690	0.1113	22.9875
	Eigen	0.1938	0.1176	0.0809	23.1301
	Anis + Lin	0.2413	0.1609	0.1083	22.8553
	Anis + Lin + Plan	0.4192	0.3843	0.2608	21.5743
	Anis + Lin + Plan + Sph	0.4825	0.4343	0.3019	20.7062
	Anis + Lin + Plan + Sph + Omni	0.5433	0.5012	0.3560	19.8065
	Anis + Lin + Plan + Sph + Omni + Sph_fit	0.5582	0.5214	0.3662	19.5343
	All_geom	0.6686	0.6582	0.4706	17.4209
Perceptual	L	0.1370	0.1235	0.0814	23.3516
	A	0.2608	0.2499	0.2450	0.2442
	B	0.1377	0.1566	0.0981	23.3771
	Curv	0.2317	0.0845	0.0613	22.9709
	sal	0.1489	0.1203	0.0876	23.4615
	L + A + B	0.4229	0.4189	0.2873	21.4179
	L + A + B + Curv	0.4628	0.5008	0.3485	21.7687
	All_perceptual	0.6132	0.6218	0.4596	21.4339
ALL	**All**	**0.9381**	**0.9362**	**0.7959**	**8.5528**

**Table 7:** Performance comparison with the state-of-the-art on the ICIP2020.

Type	Metric	ICIP2020			
		PLCC	SRCC	KRCC	RMSE
FR	PSNR [5]	0.70	0.70	-	0.82
	M-p2po [5]	0.888	0.878	-	0.522
	M-p2pl [4]	0.913	0.915	-	0.463
	H-p2po [5]	0.601	0.542	-	0.908
	H-p2pl [4]	0.649	0.602	-	0.865
	MMD [28]	0.806	0.954	-	-
	PSNRYUV [40]	0.868	0.867	-	0.564
	PCQM [3]	0.942	0.977	-	-
	GraphSIM [6]	0.890	0.872	-	0.518
	PointSSIM [37]	0.904	0.865	-	0.486
	TCDM [59]	0.942	0.935	-	0.382
RR	PCMRR [7]	0.627	0.882	-	-
NR	MVP-PCQA [43]	0.958	0.982	-	-
	Proposed	**0.9994**	**0.9962**	**0.9735**	**0.0458**

**Table 8:** Performance comparison with the state-of-the-art on the SJTU-PCQA.

Type	Metric	SJTU-PCQA			
		PLCC	SRCC	KRCC	RMSE
FR	PSNR [5]	0.2317	0.2422	0.1077	2.3124
	SSIM [5]	0.3476	0.2987	0.1919	2.1770
	PB-PCQA [51]	0.6076	0.6020	-	1.8635
	M-p2po [24]	0.8123	0.7294	0.5617	1.3613
	M-p2pl [4]	0.5940	0.6277	0.4825	2.2815
	H-p2po [24]	0.7753	0.7157	0.5447	1.4475
	H-p2pl [4]	0.6874	0.6441	0.4565	2.1255
	PSNRYUV [40]	0.8170	0.7950	0.6196	1.3151
	PCQM [3]	0.8653	0.8544	0.6586	1.2162
	GraphSIM [6]	0.8449	0.8483	0.6448	1.5721
	PointSSIM [37]	0.7136	0.6867	0.4964	1.7001
	TCDM [59]	0.930	0.910	-	0.891
	MMD [28]	0.628	0.604	-	-
RR	PCMRR [7]	0.6101	0.4816	0.3362	1.9342
NR	BRISQUE [11]	0.4214	0.3975	0.2966	2.0937
	PQA-Net [9]	0.8586	0.8372	0.6304	1.0719
	NIQE [12]	0.3764	0.2214	0.1512	2.2671
	ResSCNN [10]	0.8600	0.8100	-	-
	MVP-PCQA [43]	0.87	0.83	-	-
	MM-PCQA [60]	0.9226	0.9103	0.7838	0.7716 -
	3D-NSS [13]	0.7382	0.7144	0.5174	1.7686
	Proposed	**0.9676**	**0.9576**	**0.8454**	**0.6133**

**Table 9:** Performance comparison with the state-of-the-art on the WPC.

Type	Metric	WPC			
		PLCC	SRCC	KRCC	RMSE
FR	PSNR [5]	0.4872	0.4235	0.3080	15.8133
	SSIM [5]	0.4944	0.3878	0.3234	15.7749
	M-p2po [5]	0.4852	0.4558	0.3182	19.8943
	M-p2pl [4]	0.2695	0.3281	0.2249	22.8226
	H-p2po [5]	0.3972	0.2786	0.1943	20.8990
	H-p2pl [4]	0.2753	0.2827	0.1696	21.9893
	PSNRYUV [40]	0.5304	0.4493	0.3198	19.3119
	PCQM [3]	0.7499	0.7434	0.5601	15.1639
	GraphSIM [6]	0.6163	0.5831	0.4194	17.1939
	PointSSIM [37]	0.4667	0.4542	0.3278	20.2733
	TCDM [59]	0.807	0.804	-	13.525
	MMD [28]	0.420	0.411	-	-
RR	PCMRR [7]	0.3433	0.3097	0.2082	21.5302
NR	BRISQUE [11]	0.3155	0.2614	0.2088	21.1736
	PQA-Net [9]	0.7000	0.6900	0.5100	15.1800
	NIQE [12]	0.3957	0.3887	0.2551	22.5502
	MVP-PCQA [43]	0.85	0.86	-	-
	MM-PCQA [60]	0.8556	0.8414	0.6513	12.3506
	3D-NSS [13]	0.6514	0.6479	0.4417	16.5716
	Proposed	**0.9381**	**0.9362**	**0.7959**	**8.5528**

**Table 10:** Performance comparison with the state-of-the-art on each distortion type on ICIP2020.

Distortion		VPCC		G-PCC Octree		G-PCC Trisoup		All	
Type	Metric	PLCC	SRCC	PLCC	SRCC	PLCC	SRCC	PLCC	SRCC
FR	M-p2po [24]	0.615	0.954	0.817	0.963	0.864	0.944	0.673	0.947
	M-p2pl [4]	0.618	0.971	0.848	0.932	0.618	0.971	0.670	0.975
	H-p2po [24]	0.615	0.682	0.692	0.944	0.615	0.975	0.673	0.656
	H-p2pl [4]	0.491	0.735	0.838	0.932	0.491	0.735	0.521	0.704
	MMD [28]	0.784	0.960	0.871	0.831	0.906	0.906	0.806	0.954
	PCQM [3]	0.942	0.977	0.978	0.966	0.955	0.977	0.942	0.977
	GraphSIM [6]	-	0.855	-	0.939	-	0.770	0.890	0.872
	PointSSIM [37]	0.246	0.546	0.603	0.628	0.292	0.447	0.717	0.795
	TCDM [59]	-	0.822	-	0.885	-	0.970	0.942	0.935
RR	PCMRR [7]	0.627	0.882	0.749	0.830	0.407	0.510	0.627	0.882
NR	MVP [43]	0.958	0.982	0.987	1.000	0.957	1.000	0.958	0.982
	Proposed	**0.993**	**1.000**	**0.999**	**1.000**	**0.999**	**1.000**	**0.999**	**0.996**

**Table 11:** Performance comparison with the state-of-the-art on each distortion type on SJTU-PCQA.

Distortion		OT		CN		GGN		DS		D + C		D + G		C + G		All	
Type	Metric	PLCC	SRCC	PLCC	SRCC	PLCC	SRCC	PLCC	SRCC	PLCC	SRCC	PLCC	SRCC	PLCC	SRCC	PLCC	SRCC
FR	M-p2po [24]	0.481	0.349	-	-	0.385	0.801	0.499	0.646	0.165	0.661	0.385	0.837	0.416	0.757	0.606	0.803
	M-p2pl [4]	0.470	0.345	-	-	0.369	0.846	0.448	0.757	0.165	0.746	0.385	0.837	0.405	0.809	0.568	0.715
	H-p2po [24]	0.496	0.286	0.496	0.286	0.395	0.858	0.260	0.451	0.167	0.383	0.386	0.761	0.417	0.818	0.606	0.687
	H-p2pl [4]	0.492	0.377	-	-	0.395	0.858	0.351	0.451	0.167	0.383	0.467	0.801	0.438	0.828	0.562	0.683
	MMD [28]	0.075	0.269	0.190	0.067	0.462	0.768	0.188	0.548	0.166	0.747	0.478	0.742	0.501	0.754	0.628	0.604
	PCQM [3]	0.786	0.741	0.801	0.812	0.771	0.903	0.787	0.864	0.857	0.937	0.712	0.883	0.813	0.920	0.813	0.855
	GraphSIM [6]	-	0.693	-	0.778	-	0.916	-	0.872	-	0.886	-	0.888	-	0.941	841	0.856
	PointSSIM [37]	0.831	0.806	0.765	0.742	0.964	0.936	0.902	0.866	0.741	0.733	0.955	0.951	0.811	0.809	0.715	0.733
	TCDM [59]	-	0.793	-	0.819	-	0.921	-	0.876	-	0.934	-	0.944	-	0.951	0.930	0.910
RR	PCMRR [7]	0.271	0.279	0.014	0.029	0.187	0.175	0.398	0.428	0.093	0.006	0.509	0.430	0.265	0.132	0.263	0.219
NR	MVP [43]	0.816	0.641	0.830	0.853	**0.975**	**0.976**	**0.978**	0.927	0.966	**0.967**	0.980	0.959	0.986	0.992	0.943	0.915
	Proposed	**0.940**	**0.890**	**0.898**	**0.927**	0.964	0.972	0.886	**0.936**	**0.989**	0.854	**0.989**	**0.979**	**0.997**	**1.0**	**0.967**	**0.957**

**Table 12:** Performance comparison with the state-of-the-art on each distortion type on WPC.

Distortion		VPCC		DS		GN		G-PCC Octree		G-PCC Trisoup		All	
Type	Metric	PLCC	SRCC	PLCC	SRCC	PLCC	SRCC	PLCC	SRCC	PLCC	SRCC	PLCC	SRCC
FR	M-p2po [24]	0.684	0.697	0.779	0.900	0.686	0.728	-	-	0.534	0.464	0.399	0.566
	M-p2pl [4]	0.702	0.705	0.724	0.849	0.677	0.737	-	-	0.521	0.462	0.395	0.446
	H-p2po [24]	0.254	0.445	0.755	0.904	0.662	0.688	-	-	0.243	0.293	0.166	0.258
	H-p2pl [4]	0.377	0.558	0.614	0.861	0.664	0.692	-	-	0.299	0.355	0.226	0.313
	MMD [28]	0.734	0.790	0.688	0.786	0.826	0.863	0.032	0.062	0.509	0.502	0.420	0.411
	PCQM [3]	-	0.643	-	0.875	-	0.886	-	0.894	-	0.821	0.751	0.743
	GraphSIM [6]	-	0.612	-	0.898	-	0.840	-	0.855	-	0.816	0.856	0.841
	PointSSIM [37]	0.379	0.365	0.872	0.835	0.670	0.586	0.783	0.791	0.657	0.681	0.460	0.450
	TCDM [59]	-	0.640	-	0.882	-	0.857	-	0.795	-	0.832	0.804	0.807
	PSNR [40]	0.290	0.199	0.678	0.539	0.829	0.653	0.773	0.780	0.329	0.196	0.498	0.460
RR	PCMRR [7]	0.251	0.282	0.661	0.737	0.788	0.780	0.662	0.672	0.304	0.243	0.367	0.345
NR	MVP [43]	**0.966**	0.956	0.971	0.939	**0.999**	**1.000**	**0.995**	**1.000**	0.936	0.914	0.925	0.930
	Proposed	0.938	**0.957**	**0.995**	**0.986**	0.989	0.983	0.967	0.926	**0.975**	**0.959**	**0.938**	**0.936**

**Table 13:** Evaluation across different databases, where the label “WPC→SJTU” denotes that the model undergoes training on the WPC database and then validation using the standard testing configuration of the SJTU database.

Model	WPC→SJTU	
	PLCC	SRCC
3D-NSS [13]	0.2344	0.1817
PQA-net [9]	0.6102	0.5411
MM-PCQA [60]	0.7779	0.7693
Proposed	**0.8119**	**0.8193**

## Data Availability

http://emergimg.di.ubi.pt/icip2020PC.html; https://vision.nju.edu.cn/28/fd/c29466a469245/page.htm; https://drive.google.com/drive/folders/1dHDqKXgvkUhQdUzT7pJjrJ7zRnceFIkO (accessed on 15 May 2024).
